# Restraint and Social Isolation Stressors Differentially Regulate Adaptive Immunity and Tumor Angiogenesis in a Breast Cancer Mouse Model

**DOI:** 10.5539/cco.v6n1p12

**Published:** 2016-11-11

**Authors:** Raluca A. Budiu, Anda M. Vlad, Linda Nazario, Chandra Bathula, Kristine L. Cooper, Jessica Edmed, Premal H. Thaker, Julie Urban, Pawel Kalinski, Adrian V. Lee, Esther L. Elishaev, Thomas P. Conrads, Melanie S. Flint

**Affiliations:** 1 Department of Obstetrics, Gynecology and Reproductive Sciences, University of Pittsburgh School of Medicine, Pittsburgh, PA, 15213, USA; 2 Magee Womens Research Institute, Pittsburgh, PA, 15213, USA; 3 Department of Pharmacology & Chemical Biology, University of Pittsburgh, Pittsburgh, PA, 15213, USA; 4 Biostatistics Facility Department of Medicine, University of Pittsburgh School of Medicine, Pittsburgh, PA, 15213, USA; 5 University of Pittsburgh Cancer Institute, Pittsburgh, PA, 15213, USA; 6 University of Brighton, School of Pharmacy & Biomolecular Sciences, Brighton, BN2 4GJ, UK; 7 Department of Obstetrics and Gynecology, Division of Gynecologic Oncology, Washington University, St Louis, MO, 63110, USA; 8 Department of Surgery, University of Pittsburgh School of Medicine, Pittsburgh, PA, 15213, USA; 9 Women's Health Integrated Research Center at Inova Health System, Annandale, VA, 22003, USA

**Keywords:** breast cancer, stress, social isolation, T cells, Tregs

## Abstract

The ability of stress to induce immune suppression is widely recognized, but the mechanisms underlying the effects of stress on the adaptive immune system during tumor progression are not completely understood. To study the effect of stress on the immune system *in vivo*, we used a preclinical immunocompetent mouse model bearing 4T1 mammary adenocarcinoma cells. Mice were randomized into 4 groups, including social isolation (SI), acute restraint stress (aRRS), chronic restraint stress (cRRS), or no stress (NS). We found that SI significantly decreased the number of tumor-bearing mice still alive at the end of protocol (28 days), compared to NS mice. Although we did not detect significant changes in primary tumor volume, we observed a significant increase in the endothelial marker CD31 in primary tumors of SI mice and in lung metastases in SI and RRS mice. Survival decline in SI mice was associated with significant decreases in splenic CD8 cells and in activated T cells. From a mechanistic standpoint, RRS increased expression of *FOXP3, CXCL-10,* and *granzyme B* in mouse tumors, and the effects were reversed by propranolol. Our data demonstrate that various forms of stress differentially impact adaptive immunity and tumor angiogenesis, and negatively impact survival.

## 1. Introduction

Evidence supporting the predominant role of the immune system in cancer prognosis and clinical course of breast cancer is becoming increasingly apparent (Dieci et al., 2014; Ono et al., 2012). A successful immune response against tumors depends on effective antigen priming, robust T-cell activation and migration of effectors to target anatomical sites. Tumor infiltrating T lymphocytes (TILs) influence disease pathogenesis and the balance between various T cell subsets can often determine clinical outcome (Curiel et al., 2004). The initial observation that T cells can infiltrate ovarian tumors was made in 1982 (Haskill, Koren, Becker, Fowler, & Walton, 1982), and since then the prognostic significance of immune mediated anti-tumor effects has been well defined (Zhang et al., 2003). Triple negative breast cancer (TNBC) cases with increased effector CD8^+^ T cell infiltration are also associated with improved prognosis (Loi et al., 2014; Mahmoud et al., 2011). In contrast, increases in immune-suppressive T regulatory cells (Tregs) in primary breast tumors, through the CCR4/CCL22 axis, are indicative of poor prognosis (Gobert et al., 2009). While the CD8^+^/Treg ratio carries the most predictive value, much remains to be learned regarding the adequate selection of immune biomarkers with prognostic significance (Curiel et al., 2004; Kryczek et al., 2009; Mhawech-Fauceglia et al., 2013; Sato et al., 2005; Zhang et al., 2003).

There has been increasing interest in detailing the mechanistic role that psychological stress may play in the context of initiation, progression, metastasis, and recurrence of breast cancer. The highly-complex stress response activates both the hypothalamic-pituitary-adrenal axis and the sympathetic nervous system leading to the release of stress hormones (such as norepinephrine; NE) that positively influence carcinogenesis through mechanisms that increase proliferation, angiogenesis, and metastasis and that protect tumor cells from anoikis (Lee et al., 2009; Lutgendorf, Lamkin, Jennings, et al., 2008; Nilsson et al., 2007; Thaker et al., 2006). Adrenergic activation has been implicated as the key mediator of these effects by modulating several growth factors, such as vascular endothelial growth factor, interleukin-6, and matrix metalloproteases (Nagaraja, Armaiz-Pena, Lutgendorf, & Sood, 2013; Sood et al., 2010).

In addition to the direct effect on tumor cell biology, the negative role of chronic psychological stress on the immune system has also been characterized (Coe & Laudenslager, 2007; Segerstrom & Miller, 2004). In breast cancer, stress resulting from social isolation leads to a transient increase in CD11b+Gr-1+, CD11b+Gr-1−, and F4/80+ macrophage populations (Madden, Szpunar, & Brown, 2013) and may promote breast cancer progression through macrophage M2 polarization (Qin et al., 2015). Others have shown that stress-induced adrenergic signaling increases tumor infiltration of CD11b(+)F4/80(+) macrophages and enhances metastatic potential (Sloan et al., 2010). Evidence from ovarian cancer patients demonstrates that depression and anxiety are associated with a shift from a Th1 (cellular immunity) to a Th2 (humoral immunity) cytokine expression pattern *ex vivo* in polyclonally stimulated lymphocytes (Lutgendorfet al., 2008; Lutgendorf et al., 2005). However, the *in vivo* mechanisms by which chronic behavioral stress alters immune-effector (e.g., CD8^+^) and immune-suppressive T cells (e.g., Tregs), and thereby disease progression and recurrence, remain elusive. Furthermore, although patients with breast cancer are known to suffer considerable psychological stress throughout the disease trajectory (Bidstrup et al., 2015) individuals may experience varying levels of stressor intensity and duration. Indeed, the complex role and consequences of acute and chronic stressor-induced modulation of immune function in animals have been highlighted (Hall et al., 2014; A. W. Kusnecov & Rabin, 1994). Our primary objective in this study was to assess the role of different types of stress on the adaptive immune system in a syngeneic breast cancer model. We assessed three models of stress; a standard chronic restraint stress model; an intermittent restraint stress model; and due to evidence suggesting that social stressors may have more health relevance than physical restraint (Friedler, Crapser, & McCullough, 2015; A. W. Kusnecov, Rossi-George A, 2002; Steptoe, Shankar, Demakakos, & Wardle, 2013) we included a social isolation stress model. Because the beta-2 adrenergic receptor is believed to be the primary receptor on lymphocytes and a means through which the nervous system communicates with the immune system (Nakai, Hayano, Furuta, Noda, & Suzuki, 2014) and has been reported to prevent stress induced changes in ovarian cancer (Thaker et al., 2006) we also assessed the impact of beta blockade on immune profiles in tumor bearing mice.

## 2. Materials and Methods

### 2.1 Mice

Six-eight week old female BALB/c mice were used for the studies, as further detailed below. The animal room maintained a 12 h light-dark cycle (lights on at 6am). The mice were housed in a noise free environment and allowed to acclimate for 1 week after transport. Mice were handled daily (for approximately 5 minutes/mouse) for 2 weeks prior to the studies. Food and tap water were provided *ad libitum.* All animal protocols were approved by the IACUC at the University of Pittsburgh.

### 2.2 Syngeneic Mammary Cancer Mouse Model

Mammary 4T1 cancer cells (kindly provided by Dr. Hideho Okada) were cultured in Dulbecco's Modified Eagle's Medium with 4 mM L-glutamine and charcoal stripped bovine calf serum (10%) in a 37 °C incubator at 5% CO_2_. Syngeneic female BALB/c mice (6 weeks old; 20 ± 2 g) were purchased from The Jackson Laboratory. Mice (n = 10 per group) were injected with 1 × 10^5^ 4T1 cells/0.2mL of PBS into the left mammary fat pad. The tumors took 2 weeks to become established, with tumor volumes approx. 100 mm^3^. Tumors were measured twice weekly using a digital caliper and the tumor volumes calculated using the formula, vol (mm^3^) = L×W^2^/2; length (L, mm) and width (W, mm). Mice were randomized into one of the stress groups 3 days before treatment (day-3). At day 0, groups of mice were immediately either 1) placed individually in adequately ventilated tubes for 1 h 3 times a week (acute repetitive restraint stress, aRRS), 2) placed individually in adequately ventilated tubes for 2 h daily (chronic repetitive restraint stress, cRRS) 3) housed individually (social isolation, SI) or 4) group housed and experienced no stress (NS). All mice were sacrificed at day 28, or earlier if they experienced tumor-induced morbidity, as per IACUC regulations. All primary tumors and metastatic implants, as well as regional (inguinal and para-aortic) lymph nodes and spleens, were harvested at necropsy. All tumors were histologically confirmed by hematoxylin and eosin (H&E) staining. The spleens and regional lymph nodes were immune phenotyped using T cell markers. Protocols were considered completed at week 4 to ensure that metastasis was captured. All mice were monitored for signs of advanced disease and those considered moribund (signs of pain and distress including failure to thrive, ungroomed appearance, ruffled fur, inability to ambulate, rapid breathing/respiratory distress,rapid heart rate) were euthanized before protocol completion. Gross examination of tumors was performed at necropsy and tumors were divided in half; half were fixed in formalin for IHC and half the tissue was flash frozen in liquid nitrogen for PCR analyses. 4T1 tumors grow at the induction site (mammary fat pad) and can metastasize typically to lymph nodes, blood, liver, lung, brain, and bone (Pulaski & Ostrand-Rosenberg, 2001). Mice were sacrificed if tumor induced morbidity was detected as defined by our IACUC protocol.

### 2.3 Assessment of Psychological Stress Effects on Naïve and Activated T Cell Sub-Populations

The spleens and para-aortic lymph nodes were removed and processed into single cell suspensions (Flint et al., 2011; Flint et al., 2005) for flow cytometry. The cells were placed in RPMI media supplemented with L-glutamine, 1% penicillin/streptomycin and 10% FCS and cell viability was assessed by trypan blue dye exclusion. Cells were 95–98% viable for all experiments.

### 2.4 Analysis of Cell Activation by Flow Cytometry

Multi-parameter flow cytometry was used to determine T cell subpopulations and their activation status. Cells were stained with anti-mouse CD3-FITC or -PerCP (clone 145-2C11, Miltenyi Biotec), CD4-PacificBlue (clone RM4-5), CD8-APC-Cy7 (clone 53-6.7), CD69 – PE (clone H1.2F3) and anti-mouse Foxp3-APC (clone MF23) (BD Biosciences). All antibodies were diluted according to manufacturers’ instructions. Gating was set using an isotype-matched control antibody (Supplementary Figure 2). Stained cells were analyzed on a LSR II flow cytometer using the FACSDiva data analysis software (BD Biosciences).

### 2.5 Analysis of Tumor Microvasculature

Formalin-fixed, paraffin-embedded mammary tumors and lungs were cut in 5-µm-thick transverse sections and stained with H&E. For immunohistochemistry, tissue sections were deparaffinized and rehydrated in serial ethanols. Antigen retrieval was performed with Tris EDTA (pH 9.0) buffer with boiling. After permeabilization with 0.3% Triton X-100 for 10 min and blocking with 2% BSA for 20 min, tissue sections were immunostained for CD31 expression (1:50; Abcam), an endothelial cell marker and marker of tumor angiogenesis. Sections were then rinsed with PBS and with IHC select secondary anti HRP detection set (Millipore, UK). Staining was microscopically visualized and the number of CD31^+^ vessels were scored in 10 optical fields per tumor sample.

### 2.6 Analysis Of Cytokine mRNA in Tumor Tissue

The tissue was thawed and mRNA was isolated using the Qiagen RNAeasy mini kit (Qiagen) as per manufacturers’ instructions. RNA concentration was confirmed using a Bioanalyzer 2100 (Agilent Technologies, Palo Alto, CA) and cDNA was made from 1 µg of total RNA using the first strand cDNA synthesis kit (SABiosciences, Frederick, MD). One microgram of cDNA used for real-time PCR analysis (ABI PRISM 7700, Applied Biosystems Inc., Waltham, MA) utilizing the primer probe sets with the Fam dye (Applied Biosystems) (*Foxp3, CCL22, Granzyme B, CCL5, IFN*-γ, *CXCL9, CXCL10, CXCL11* and *IFN*-γ). Data analysis was carried out with the instrument software and the ΔΔCt method was used with normalization of the raw data to an external RNA control.

### 2.7 Assessing the Effects of Beta Blockade Early in Mammary Tumor Development

We next designed a pilot study to understand the mechanisms of how daily stress can influence the adaptive immune system early on in tumor development. Mice with 4T1 mammary tumors reaching 100 mm^3^ (which occur within 5 days following injection; n = 3/group) were placed into two groups; 1) restraint stress (2 hour stress applied daily for 3 days at 10am) or no stress controls. Each group was treated with either propranolol (10 mg/kg IP every day for 3 days), or PBS (controls). At necropsy, all tumors and spleens were harvested and immune phenotyped by flow cytometry and cytokine expression assessed by real time PCR as described above.

### 2.8 Statistical Analyses

Tumor volume measurements were log-transformed to satisfy linearity assumptions and a mixed model was fitted to account for correlation of repeated measures within subjects. Growth rates, represented as slopes, were compared across and within groups. The proportion of mice alive in each group at the final time point of the experiment was compared to control using the Fisher’s exact test. We did not use Kaplan Meier curves or log-rank testing because animals were sacrificed. Continuous measurements taken at the time of sacrifice, including tumor weight, tumor micro-vessel density and flow cytometry markers, were compared to controls using a cell means model. Changes from baseline, in mouse weight, were compared to controls using a repeated measures analysis of variance model. The number of metastatic sites in each group was compared to controls using a Poisson general linear regression model. Real-time PCR data was normalized to the reference gene and differences between treatment and control groups were compared across stress groups using a cell means model and general linear hypothesis tests. All tests were two-sided at a nominal 0.05 significance level and standard deviations are represented in all plots.

## 3. Results

### 3.1 Social Isolation (SI) Stress Significantly Reduces Survival in Mammary Tumor-Bearing Mice

**Figure 1:**
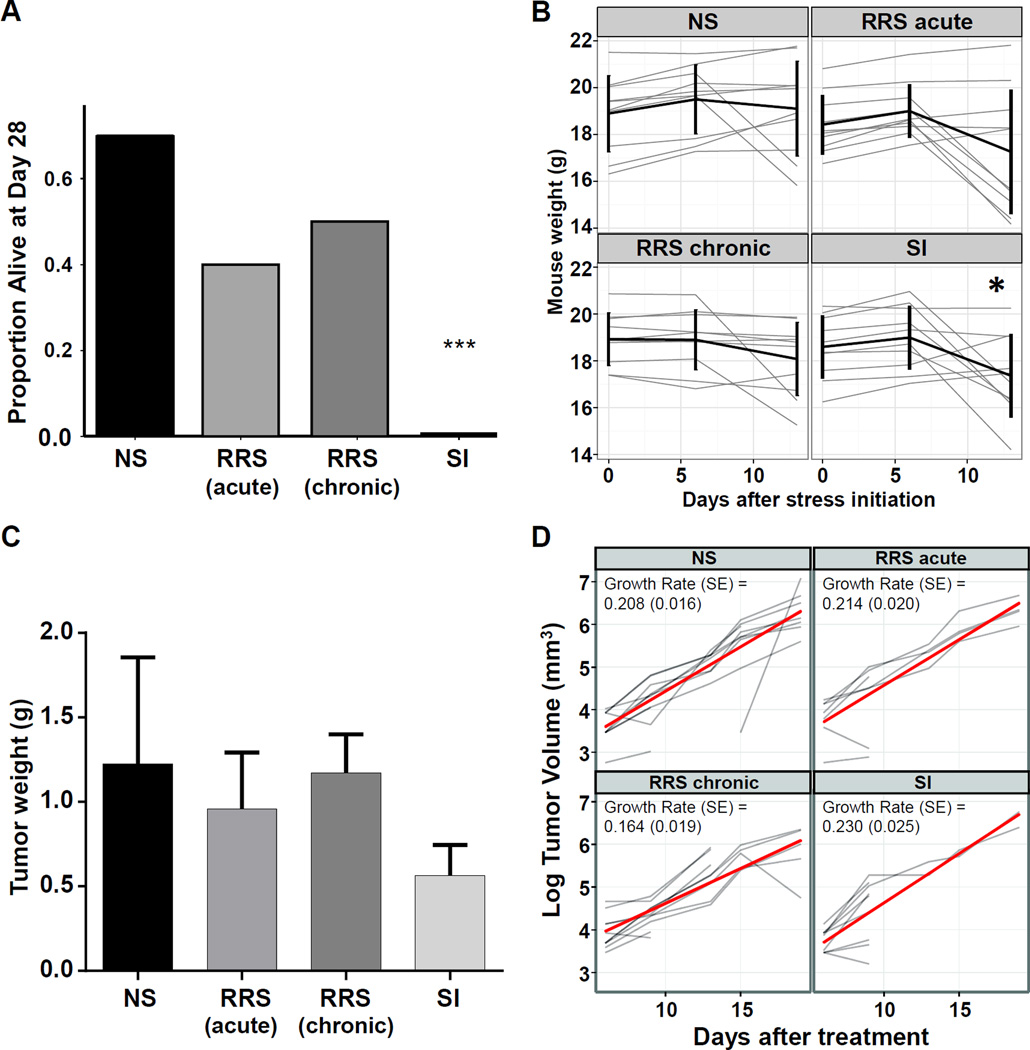
Social isolation (SI) decreases the proportion of 4T1 mammary tumor bearing mice alive at the end of protocol

To establish if different psychological stressors can affect the survival of tumor bearing mice, we examined the impact of three different stress regimens: aRRS (one hour thrice weekly), cRRS (two hours daily), or SI (mice separated immediately after the tumor reached 100 mm^3^) in the 4T1 syngeneic mammary carcinoma model. We determined that SI significantly decreased the number of tumor-bearing mice still alive at the end of protocol, compared to NS mice (Figure 1A. *p* = 0.003). Mice subjected to either acute or chronic RRS did not significantly impact survival to the scheduled end of protocol at day 28 (Figure 1A). Social isolation significantly decreased overall body weight, a common symptom of stress, on day 13 compared to day 0 (Figure 1B; *p* = 0.047) whereas aRRS or cRRS did not significantly influence the overall body weight during disease progression in either the mammary (Figure 1B). Furthermore, there were no significant differences in primary mammary tumor weights at the time of sacrifice (Figure 1C, Supplementary Figure 1). Considering that the mammary tumors were weighed at the time of sacrifice and 7/10 mice in the SI group were sacrificed before day 10 rather than the scheduled end of protocol (day 28), it appears that the primary tumors are more aggressive in this group. Stress did not significantly alter mammary tumor volume (Figure 1D).

Mice injected with 4T1 tumor cells in the mammary fat pad were randomly assigned to the following treatment groups: SI, cRRS and aRRS (n = 10/group). Non-stressed animals (NS, n = 10/group) were kept as controls. (A) Proportion of mice surviving at the end of protocol. Statistical significance was determined using the Fisher’s exact test. (B) Mice were weighed prior to tumor implantation and monitored weekly throughout the study. Black line represents mean ± SD. Changes from baseline in mouse weight were compared to control using a repeated measures analysis of variance model * *p* < 0.05. (C) Tumor weights at necropsy. Tumor weights were compared to controls using a cell means model. (D) Tumor volumes in mice with 4T1 mammary tumors were compared on a log scale using a linear model for repeated measures. Red line is mean growth rate *** *p* < 0.001.

### 3.2 Stress increased CD31 expression but not the number of metastatic sites in mice with mammary tumors

**Figure 2:**
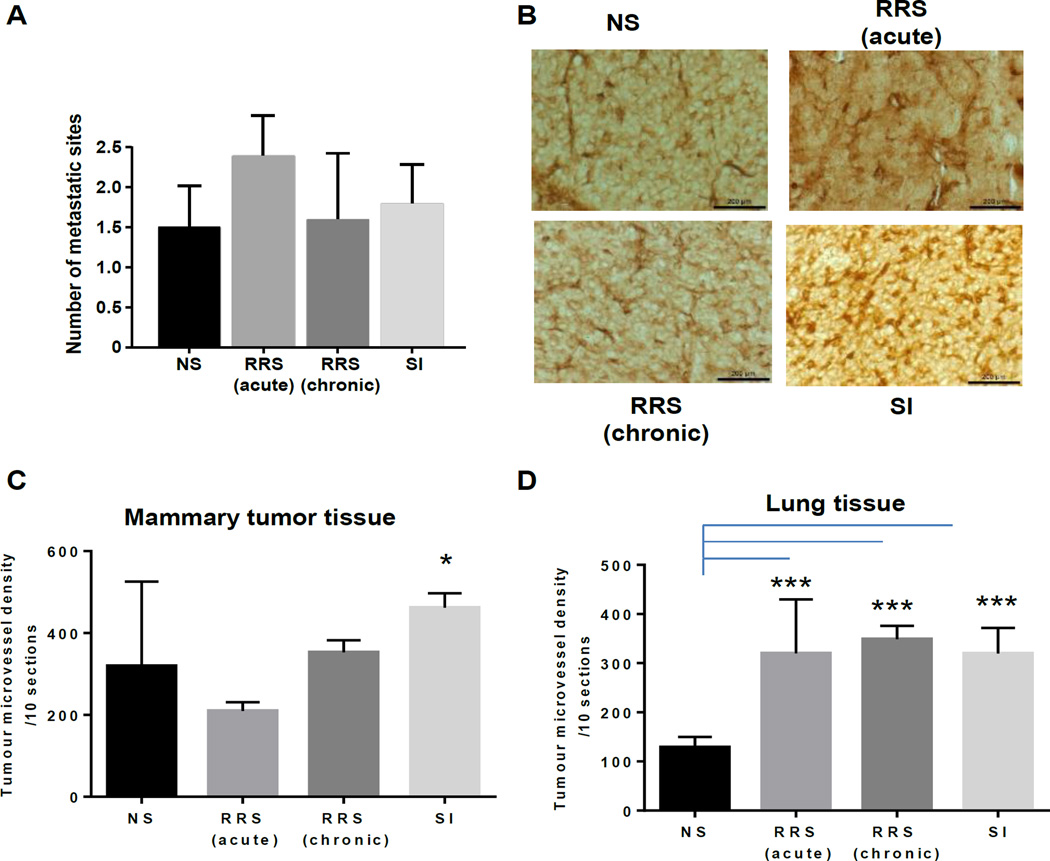
Stress has no effect on the average number of metastatic sites per mouse with mammary tumors but increases CD31 expression (A) Average number of metastatic sites/mouse in mammary tumor bearing mice. At the time of necropsy, metastasis was examined by gross necropsy and validated by a pathologist (E.E.). (B) CD31 expression detected by IHC in mammary tumors obtained at necropsy from all four mouse groups. (C, D) Tumor micro-vessel density in mammary tumor, and in lung metastases, respectively. Tumor micro-vessel densities were compared to controls using a cell means model. The number of metastatic sites in each group was compared to control using a Poisson general linear regression model. * *p* < 0.05 ** *p* < 0.01, *** *p* < 0.001

To establish if different stressors alter metastasis, we performed a gross examination at necropsy followed by histopathology assessments of fixed tissues by a pathologist (Figure 2A and Table 1). Over 50% of mice in each group developed metastases to the lungs, although other anatomical sites (liver, intestine, spleen, ovary, diaphragm, lymph nodes, adrenal glands and kidney) that harbored metastatic implants were also detected. We found no significant difference in the number of metastatic implants between the stressed groups however we did observe a significant increase in the numbers of mice with metastasis in the aRRS group. To determine if different stressors impact angiogenesis we next assessed CD31 expression in mammary tumor tissue. CD31 can be used to demonstrate the presence of endothelial cells and evaluate the degree of tumor angiogenesis. We found that there was a significant increase in CD31 in SI mice compared to NS mice (*p* = 0.02; Figure 2B, C) indicating an increases in angiogenesis and rapidly growing tumors. In the lung, there was a significant increase in CD31 expression in all three stressed groups when compared to NS controls (*p* < 0.001 in all groups; Figure 2D).

### 3.3 Stress Differentially Affects the Number and Activation Status of T Lymphocytes in Mice with Mammary Tumors

**Figure 3:**
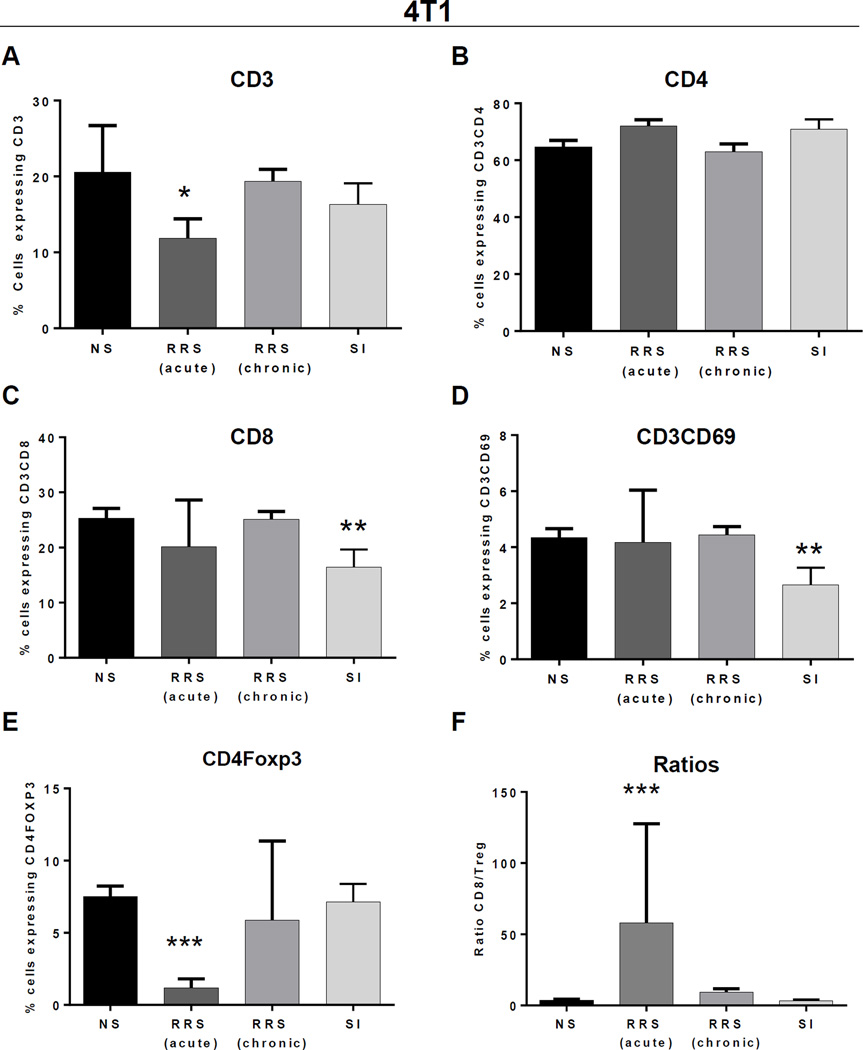
Stress differentially affects the number and activation of immune cells in spleens of 4T1 mammary tumor bearing mice Splenocytes were isolated from each group of tumor bearing mice and the percentages of cells expressing CD3^+^ (A) CD3^+^CD4^+^ (B), CD3^+^CD8^+^ (C), CD3^+^CD69^+^ (D) and CD4^+^Foxp3^+^ (E) were assessed using multicolor flow cytometry and FACSDiva software. (F) Splenocyte CD8 to CD4^+^Foxp3^+^ Treg ratio. Flow cytometry markers, were compared to controls using a cell means model. * *p* < 0.05 ** *p* < 0.01, *** *p* < 0.001.

**Figure 4:**
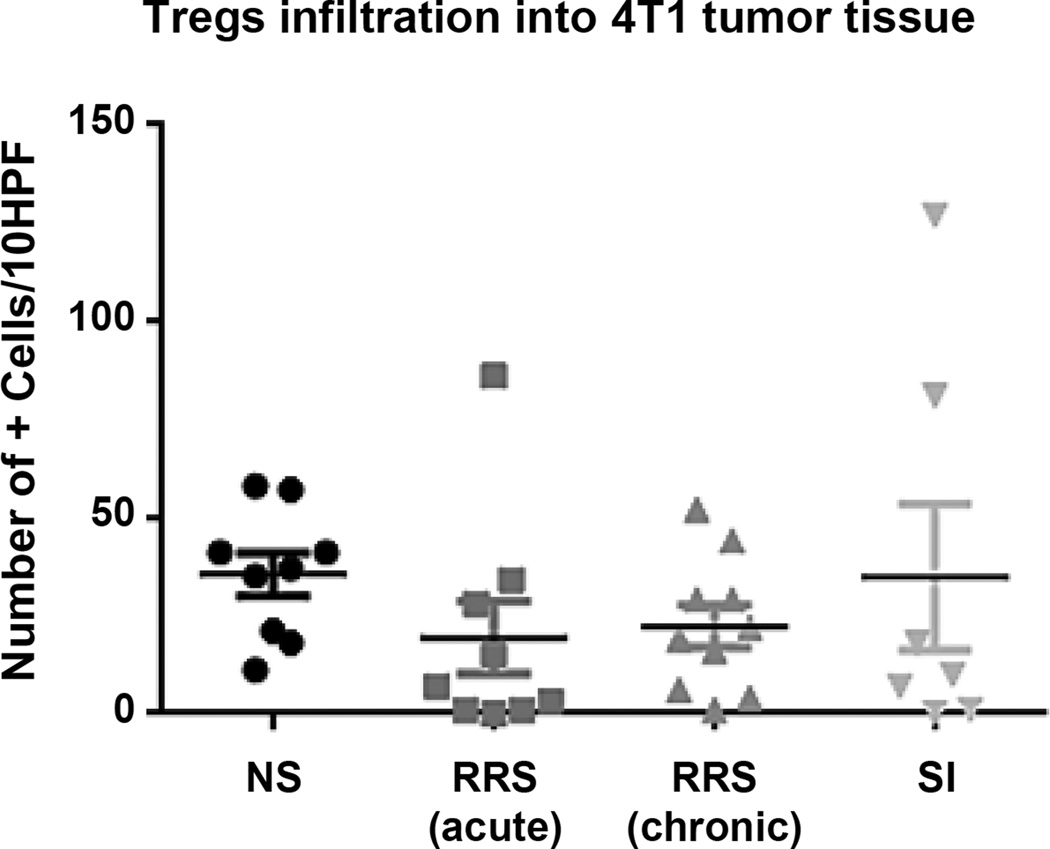
Stress did not alter Treg infiltration in the tumor microenvironment Tumor sections were stained by IHC for Foxp3 protein and mammary tissue infiltrating positive cells were counted as explained in Materials and Methods. Lines represent mean value.

To explore changes in systemic immunity, we examined the effects of various stressors on the composition and activation status of immune cells isolated from spleens of mammary tumor-bearing mice (Figure 3). Acute RRS induced a significant decrease in CD3^+^ cells (Figure 3A p = 0.009). Although we found no significant changes in CD4^+^ T cells by stress (Figure 3B), SI led to significantly lower percentages of CD3^+^CD8^+^ T cells (p = 0.012; Figure 3C) and of activated CD69^+^CD3 (p = 0.018) T cells (Figure 3D). Acute RRS was the only stressor that triggered changes in Foxp3^+^ Treg accumulation, leading to a significant decrease of Treg (p < 0.001) in mouse spleens (Figure 3e) and increase in the ratio of CD8^+^/Tregs in aRRS exposed mice (Figure 3f). None of the measured immune parameters were significantly affected by cRRS. To further understand the tumor micro-environment, we assessed the presence of tumor-infiltrating Tregs, typically considered negative indicators of breast cancer prognosis (Gobert et al., 2009). We found no statistical difference across the different stress groups (Figure 4).

### 3.4 Propranolol Alters the Immune Profile in the 4T1 Mammary Tumor Model

**Figure 5:**
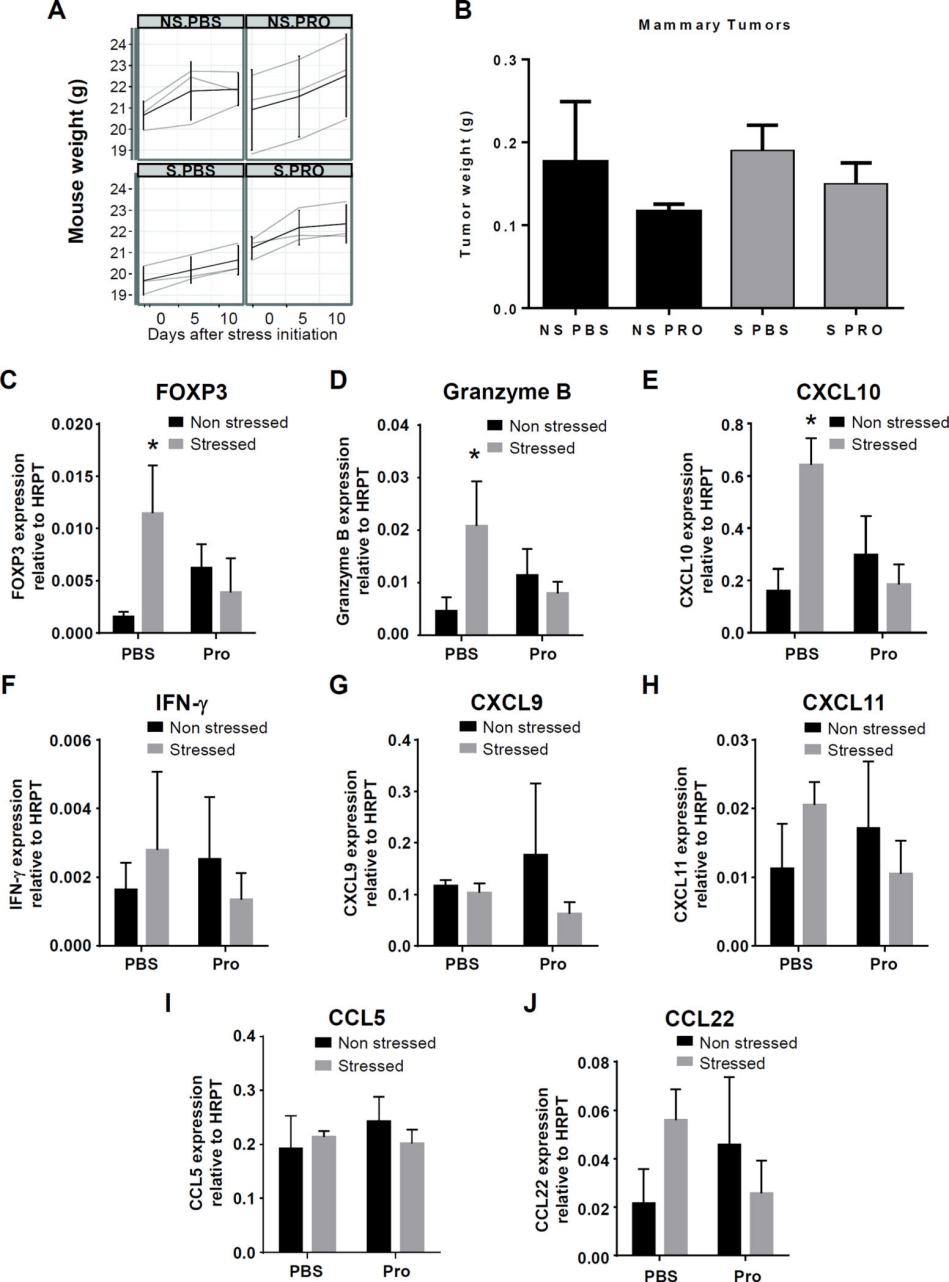
Acute stress and propranolol affects immune patterns in 4T1 mammary tumor bearing mice (A) Mice with 100 mm^3^ mammary tumors were placed into two groups; aRRS or NS (controls). Each group was treated with either propranolol (PRO, 10 mg/kg IP every day for 3 days), or PBS as controls. Mice were weighed and monitored throughout the study. Each line represents one mouse and changes from baseline, in mouse weight, were compared to control using a repeated measures analysis of variance model. (B) Fresh tumor samples were weighed and isolated from stressed (S) and control (NS) mice; (C–J) Gene expression analyses via qRT-PCR, using tumor-extracted RNA and primers for *FOXP3* (C) *granzyme B* (D) *CXCL-10* (E) *IFN*-γ (F) *CXCL9* (G), *CXCL11* (H), *CCL5* (I) and *CCL22* (J). Real-time PCR data was normalized to the reference gene and differences between treatment and control were compared across stress groups using a cells means model and general linear hypothesis tests. * *p* < 0.05.

To understand the role of the beta-2 adrenergic receptor on the immune system in tumor bearing mice, we further investigated the effects of acute stress on *in vivo* tumor biology early in tumor development in the presence/absence of a beta blocker in RRS and NS mice with 4T1 mammary tumors. Previous research suggests that 4T1 mammary adenocarcinoma cells lack functional α- and β-AR, and are unresponsive to NE (Szpunar, et al., 2013). This allowed for the examination of the influence of NE on T cells rather than on the tumor cells directly. We found no significant effects on body weights (Figure 5A) or significant differences in Tregs in the spleen (data not shown), likely due to the short duration of the study. However, we observed a trend towards a decrease in the primary tumor weights in mice treated with propranolol in both the NS and daily RRS groups (Figure 5B).

To test the effects of beta blockers on the chemokine environment in tumors of RRS-exposed animals, we examined the expression of genes that are characteristic of either immune suppression (*Foxp3, CCL22*) or effector anti-tumor immunity (*Granzyme B, CCL5, IFN*-γ, *CXCL9, CXCL10* and *CXCL11*) (Figure 5C–J). We observed that RRS induced significant increases in gene expression of *Foxp3* (*p* = 0.017, Figure 5C), the effects of which were abrogated by propranolol. RRS also significantly increased expression of *granzyme B,* a serine protease found in the cytoplasm of cytotoxic T cells and NK cells *(p* = 0.033, Figure 5D*)* and of *CXCL10 (IP-10),* a T cell chemoattractant, (*p* = 0.038, Figure 5E), both of which were reduced by propranolol. In contrast, we did not observe significant changes in *IFN*-γ, *CXCL9, CXCL11, CCL5 or CCL22* (Figure 5F–J). Overall, these results suggest that stress may induce both effector and suppressive chemokines and that propranolol counteracts these effects.

## 4. Discussion

In this study, we demonstrated that the duration and type of psychological stressors can differentially impact the adaptive immune system in an immunocompetent mouse model of triple negative breast cancer. This is the first report showing the side-by-side comparison of acute RRS, chronic RRS and SI on T cells. Using the 4T1 cells to model aggressive, triple negative breast cancer, we show that SI significantly increased disease progression resulting in an early sacrifice of the mice. SI has previously been associated with increased mammary tumor growth and tumor invasiveness in SV40 T-antigen FVB/N (TAg) mice, and in mouse and rat xenografts with MDA-MB-231 cells (Hermes et al., 2009; Madden et al., 2013; Williams et al., 2009). However, the effects of stress on tumor size were thought to have a short term impact and were dependent on when mice were subjected to SI (i.e. before or after palpable tumor formation) (Madden et al., 2013). We did not observe significant effects of SI on the primary tumor size at the time of death which raises the possibility that SI modulates the ability to adapt to growing tumors, presumably through the immune system, rather than affecting the ability to control tumor growth. Much work on the effects of stress has focused on suppressive effects of innate immunity, carried out by granulocytes, macrophages, and NK cells (Duggal, Upton, Phillips, Hampson, & Lord, 2015; Qin et al., 2015; Varker et al., 2007). Nevertheless it is the adaptive immunity involving T and B cells that is ultimately responsible for the highly sensitive, antigen-specific mechanisms leading to robust cytotoxicity against tumors. Our findings show that SI preferentially impacts CD8^+^ T cell numbers and activation, as evidenced by decreases in CD8^+^ and CD3^+^CD69^+^ T cells. These findings indicate that, even in advanced stages of tumor progression, stressors can regulate the adaptive immune response. We also report that stress, independent of type, does not significantly increase the number and location of metastatic sites in our mammary tumor model compared with the NS groups, most likely due to the aggressive *in vivo* behavior inherent to the 4T1 tumors. Sloan *et al.* have shown an increase in breast cancer metastasis using a 66cl4 breast cancer Balb/c mouse model subjected to chronic restraint stress (Sloan et al., 2010). In contrast to our study, the stress was applied prior to tumor inoculation and the tumor model is not reportedly as aggressive as the short-lived 4T1 model employed here. It is likely that if the mice carried slower growing primary tumors, more metastatic sites may have been identified. Further we did observe an increase in the number of mice demonstrating metastasis in the aRRS group. Nevertheless, the significantly increased CD31^+^ vascular expression in the mammary tumors (with SI) and lung suggests that angiogenesis, a key process in tumor development, may be regulated by stress and may be a plausible explanation for the reduced survival observed in SI mice. Although tumor cells were originally thought to be the main promotors of tumor angiogenesis, researchers have demonstrated that the tumor microenvironment and infiltrating immune cells such as T cells can regulate the process of tumor angiogenesis (Stockmann, Schadendorf, Klose, & Helfrich, 2014). It is possible that stress can impact the immune microenvironment leading to this enhanced angiogenesis. To further understand the role of adaptive immunity in the tumor tissue, we investigated the impact of RRS on tumor weight and on immune suppression markers (*Foxp3, CCL22*), as well as markers for anti-tumor immunity (*Granzyme B, CCL5, IFN*-γ, *CXCL9, CXCL10* and *CXCL11*). RRS significantly increased Foxp3 expression in Tregs in tumors, which was abrogated by propranolol. This finding is of particular importance given the previous reports showing that increases in Tregs in primary mammary tumors, through the CCR4/CCL22 axis, are indicative of poor prognosis (Gobert et al., 2009). Finally, we show that RRS strikingly elevated *CXCL10* in tumor tissue, an effect reversed by propranolol. CXCL10 was initially thought to be a chemoattractant for T cells (Taub et al., 1993); however, more recently its role in breast cancer is thought to be more complex. It has been reported that CXCL10 may act in a paracrine fashion affecting the peritumoral CD4^+^ and CD8^+^ lymphocytes, and in an autocrine fashion contributing to tumor migration and progression (Mulligan et al., 2013). RRS also significantly induced *granzyme B,* a major component of cytolytic T and NK cell function (Alizadeh et al., 2014; Wendel, Galani, Suri-Payer, & Cerwenka, 2008). Granzyme B is found to be expressed in breast carcinomas (Hu et al., 2003) and thought to be either protective or work as an immune regulator. More recent reports suggested that granzyme B may contribute to suppression of anti-T cell responses (Lindner et al., 2013) and may be highly expressed in Tregs in the tumor microenvironment (Li et al., 2011). Further mechanistic work to assess the relative levels of each T cell subset in the breast and distant metastatic sites isolated from non-stressed and stressed, and socially isolated mice is ongoing in our laboratory. Our findings that RRS increases *CXCL10, granzyme B* and Tregs in the early stages of tumor progression may contribute to our understanding of the mechanisms of stress on tumor progression and demonstrate that the use of beta-blockers may serve to reverse the stress-induced impact on the adaptive immune system and mitigate cancer progression. It would be interesting to translate our findings in syngeneic models into patient derived xenografts which maintain characteristics of the primary tumor in the patient. In conclusion, we demonstrate that various forms of stress differentially impact anti-tumor adaptive immune responses, angiogenesis and mortality in a pre-clinical mouse model. In view of the increasing evidence supporting the predominant role of the immune system in cancer prognosis and that beta blockers are currently being considered for treatment of cancer, dissecting the complexity of stress on the immune system in the tumor microenvironment is needed.

## Supplementary Material

01

## Figures and Tables

**Table 1:** Metastatic profile of 4T1 tumors according to stress.

	Primary tumor	Lung	Liver	Spleen	Ovary	Diaphragm	Intestine	Lymph nodes	Pancreas	Adrenal gland	Kidney	Total
NS	10	7	2	1	0	0	0	2	1	1	1	25
RRS (acute)	10	5	3	3	1	3	2	2	3	0	2	34
RRS (Chronic)	10	7	0	1	1	1	1	1	1	1	1	25
SI	10	4	4	0	1	3	2	2	0	0	0	26

The numbers represent mice within each group with metastases present at the listed anatomic sites. The total value is the sum of all metastatic sites within each group. A total of n = 10 mice were injected in each of the four groups. NS; no stress.
