# Supplementary material for: Restraint and Social Isolation Stressors Differentially Regulate Adaptive Immunity and Tumor Angiogenesis in a Breast Cancer Mouse Model
Source: Cancer Clin Oncol. Author manuscript; Available in PMC 2017 Jun 8. (PMC5464739; doi:10.5539/cco.v6n1p12)
Supplement: 01 [file NIHMS833342-supplement-01.pdf]

Supplementary Figure 1

Tumor weights in mice with 4T1 mammary tumors

Tumor weights	NS	aRRS	cRRS	SI
1	1.75	0.11	1.03	no data
2	1.49	0.11	2.18	0.42
3	0.94	0.14	2.36	0.45
4	1.32	0.32	1.28	0.13
5	1.33	2.1	0.69	0.19
6	1.32	0.14	0.96	0.11
7	0.19	**2.1**	1.39	0.57
8	1.9	1.9	0.17	0.33
9	1.87	2.57	0.26	1.18
10	0.13	0.11	1.39	1.71

Supplementary Figure 2

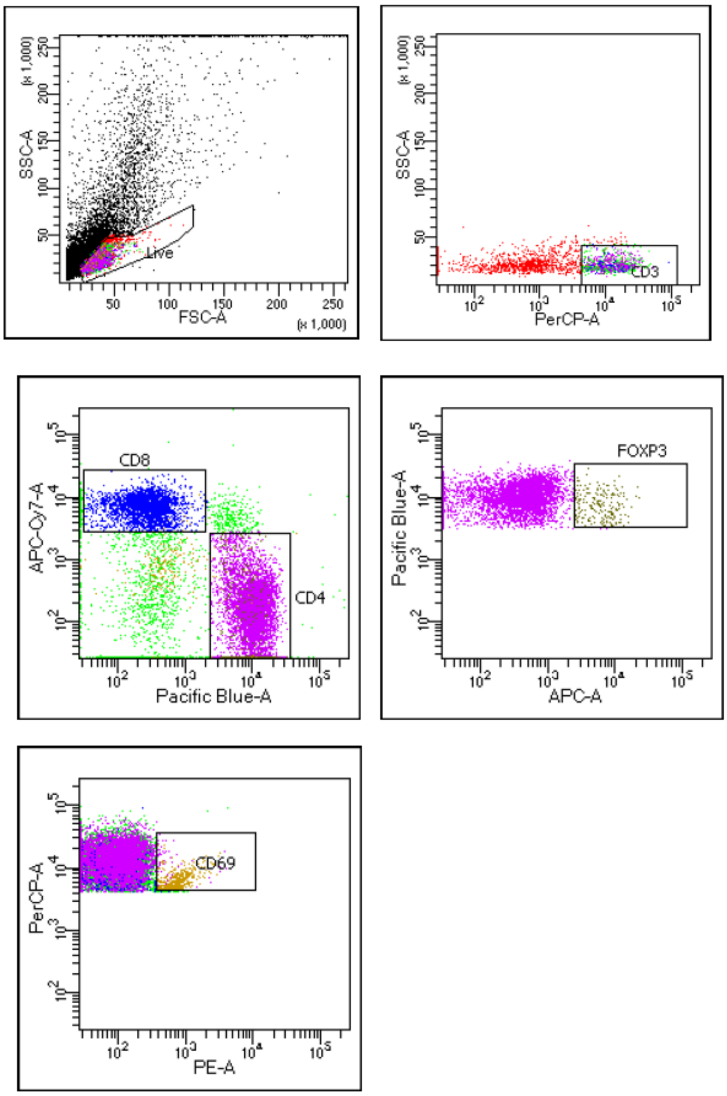

Figure 1. Representative dot plots from a NS mouse

Copyrights

Copyright for this article is retained by the author(s), with first publication rights granted to the journal.

This is an open-access article distributed under the terms and conditions of the Creative Commons Attribution license (http://creativecommons.org/licenses/by/4.0/).
